# Antioxidant, Antimicrobial, Cytotoxicity, and Larvicidal Activities of Selected Synthetic Bis-Chalcones

**DOI:** 10.3390/molecules27238209

**Published:** 2022-11-25

**Authors:** Aswathi Moothakoottil Kuttithodi, Divakaran Nikhitha, Jisha Jacob, Arunaksharan Narayanankutty, Manoj Mathews, Opeyemi Joshua Olatunji, Rajakrishnan Rajagopal, Ahmed Alfarhan, Damia Barcelo

**Affiliations:** 1 Molecular Microbial Ecology Lab, PG and Research Department of Zoology, St. Joseph’s College (Autonomous), Devagiri, Calicut 680 555, Kerala, India; 2 Division of Cell and Molecular Biology, PG and Research Department of Zoology, St. Joseph’s College (Autonomous), Devagiri, Calicut 673 008, Kerala, India; 3 PG and Research Department of Chemistry, St. Joseph’s College (Autonomous), Devagiri, Calicut 673 008, Kerala, India; 4 African Genome Center, Mohammed VI Polytechnic University, Ben Guerir 43150, Morocco; 5 Department of Botany and Microbiology, College of Science, King Saud University, P.O. Box 2455, Riyadh 11451, Saudi Arabia; 6 Water and Soil Research Group, Department of Environmental Chemistry, IDAEA-CSIC, Jordi Girona 18–26, 08034 Barcelona, Spain

**Keywords:** bis-chalcones, antioxidant activity, antimicrobial activity, larvicidal activity, chemical structure

## Abstract

Plants are known to have numerous phytochemicals and other secondary metabolites with numerous pharmacological and biological properties. Among the various compounds, polyphenols, flavonoids, anthocyanins, alkaloids, and terpenoids are the predominant ones that have been explored for their biological potential. Among these, chalcones and bis-chalcones are less explored for their biological potential under in vitro experiments, cell culture models, and animal studies. In the present study, we evaluated six synthetic bis-chalcones that were different in terms of their aromatic cores, functional group substitution, and position of substitutions. The results indicated a strong antioxidant property in terms of DPPH and ABTS radical-scavenging potentials and ferric-reducing properties. In addition, compounds **1**, **2**, and **4** exhibited strong antibacterial activities against *Escherichia coli, Pseudomonas aeruginosa, Staphylococcus aureus*, and *Salmonella enteritidis*. The disc diffusion assay values were indicative of the antibacterial properties of these compounds. Overall, the study indicated the antioxidant and antimicrobial properties of the compounds. Our preliminary studies point to the potential of this class of compounds for further in vivo investigation.

## 1. Introduction

Plants are an important component of the biosphere that is essential for the sustainability of entire ecosystems [1]. They act as the primary source of food for other organisms, which are primarily composed of carbohydrates [2]. Apart from that, these plants are also home to a wide variety of compounds. Phytochemicals are important components obtained from plants by various methods and they are used as food and medicinal compounds by various populations in India and around the world [3]. Most compounds of plant origin are useful for their biological and pharmacological properties. [4]. A majority of these are allelochemicals that are known to repel various pests that attach to these plants [5,6]. Various biochemicals such as flavonoids, alkaloids, saponins and phenolic compounds are a useful part of medicine [7]. Compounds are extracted from almost all parts of a plant including the root, bark, flower, leaf, etc. Fatty oils, both essential and non-essential, and many active compounds can be seen in different parts of a single plant. Some produce medicinal effects whereas some produce toxic effects.

Plant products including primary and secondary metabolites are widely applied in the pharmacological field and nutritional aspects. These biochemical products are “side-tracks” or secondary metabolites that are essential in plant growth and development, protection, attraction or signaling. The main chemical groups of bioactive compounds in plants include polyphenols, flavonoids, anthocyanins, tannins, and chalcones. Flavonoids are an important class of bioactive compounds of plant origin. Flavonoid compounds possess antioxidant, antimicrobial, antiviral, and antitumor activities. The intake of large amounts of flavonoids can help prevent cancer and heart disease. Structurally, they are further classified into chalcones, flavones, isoflavones, flavanols, and anthocyanins. A chalcone is a compound that consists of two aromatic rings linked by an unsaturated 𝛼, 𝛽-ketone. Chalcones are a class of bioactive plant metabolites that are equipped with numerous biological and pharmaceutical benefits to humans. Plants belonging to the Leguminosae, Asteraceae and Moraceae families are rich in natural chalcones [8,9]. The radical-quenching abilities of the chalcones isolated from different plants have been widely reported [10,11]. The *Angelica keiskei*-derived chalcones have been shown to block the activities of cysteine proteases of the COVID virus [12]. A pharmacological analysis has indicated the antiradical and anti-edematous properties of these *A. keiskei* chalones [13]. Later, studies by Shin et al. [14] found that *A. keiskei* chalcones inhibit cytokine production in macrophages. Further, Enoki et al. [15] indicated that 4-hydroxyderricin (4-HD) and xanthoangelol, the two major chalcones in the plant, inhibit the development of diabetes via peroxisome-proliferator-activated receptor-gamma activation in mice. Likewise, the extracted chalcones from different species of *Artocarpus* inhibited platelet aggregation in vitro [16]. Ngameni et al. [17] indicated the potential of *Dorstenia turbinata* chalcones to inhibit brain tumor cell proliferation and invasion by blocking MMP2. The bioavailability of chalcones, i.e., the proportion of chalcone that enters the circulation when introduced into the body and its ability to have an effect, is low. The issue itself has important implications for the pharmaceutical applications of chalcones or their derivative molecules. [18]. Structurally, dibenzylidines or bis-chalcones also belong to the chalcone family [19]. Synthetic chalcones and bis-chalcones have therefore become more important than the naturally present chalcones. However, the medical properties of bis-chalcones are less known. The chalcones are also known for the inhibition of microbial populations, especially bacteria and fungi [20,21]. Further, these compounds are known to inhibit diseases associated with oxidative damage and inflammation [22].

**Figure 1:**
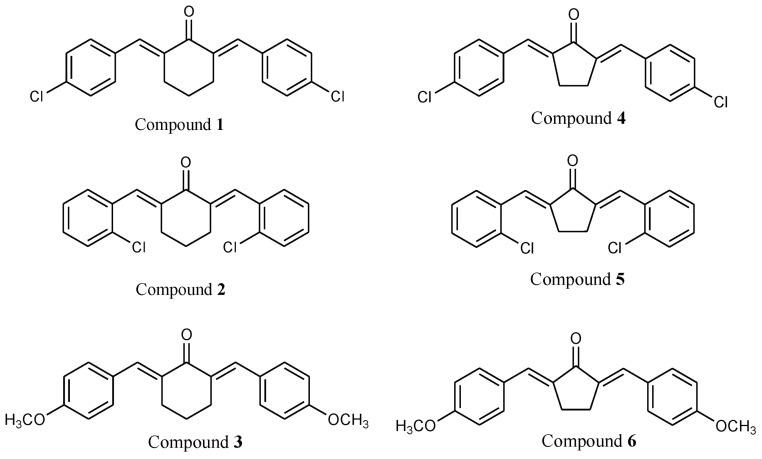
Molecular structures of synthetic chalcones **1** to **6**.

Plant chalcones are flavonoid derivatives formed in the biosynthetic pathway of flavonols. They do not accumulate in plants in larger quantities; hence, the availability of these compounds is too limited from plant sources. Another challenge that limits the potential of chalcones is their low half-life. In this work, we synthesized six bis-chalcone compounds whose structures are shown in Figure 1. Compounds **1** to **3** are cyclohexanone derivatives while compounds 4 to 6 are cyclopentanone derivatives. We report the properties of these synthetic bis-chalcones as antioxidant molecules and antibacterial and larvicidal agents. 

## 2. Results and Discussion

### 2.1. Characterization of Compounds

Phytochemicals are important compounds that are present in different plant parts at different concentrations [23]. These compounds are produced as part of the metabolic pathway or as secondary metabolites. Chalcones are one of the few bioactive compounds in plants [18]; they are known to have strong pharmacological activities against infectious and chronic diseases [24]. However, these molecules have a lower biological availability in humans. To overcome this issue, various synthetic chalcones/bis-chalcones have been chemically synthesized and studied [25,26]. In the present study, we evaluated six synthetic bis-chalcones for their biological efficacies. The synthetic chalcones were characterized by FT-IR and NMR spectroscopy techniques and high-quality figures are included in Supplementary Figures S1–S13.

### 2.2. Antiradical Potentials of Various Synthetic Bis-Chalcones

The synthetic bis-chalcones exhibited strong antioxidant activity; the scavenging of DPPH, nitric oxide, and ABTS radicals was high in compound **1** and compound **2**, whereas compounds **5** and **6** were the least active (Table 1). The FRAP assay identified the EC50 values of compounds A, B, and C; the highest activity was shown by compound A (EC50 of 1.35 ± 0.10 µg/mL). This was followed by compound B (5.24 ± 0.21 µg/mL) and compound C (12.4 ± 0.20 µg/mL). The results indicated the antioxidant properties of the selected bis-chalcones, especially for compounds **1**, **2**, and compound **4**. As can be seen from the structure, all of these molecules have the –Cl group as a substitution in their aromatic core unit. Strong radical-scavenging, reducing and enzyme inhibitory properties of these molecules can be attributed to the presence of the chlorine substitution in these bis-chalcones. Our results showed the ability of bis-chalcones to strongly inhibit the DPPH radical and reduce ferric ions. Antioxidant abilities eventually help to reduce oxidative radicals from various biological systems and thereby prevent the development of oxidative stress [27,28]. Oxidative damage induced by free radicals results in the progression of degenerative disorders including diabetes, obesity, cardiovascular problems, and neoplasia [29,30]. Hence, synthetic bis-chalcones may be helpful to prevent the development and progression of various oxidative-stress-associated diseases.

### 2.3. Synthetic Bis-Chalcones as Antimicrobial Agents

The selected synthetic bis-chalcones exhibited varying toxicity against the bacterial strains (Table 2); compound **2** was the most active against *Escherichia coli* (22.5 ± 0.2 mm). Similarly, compound **2** and compound **4** had LC50 values of 57.6 ± 3.2 and 69.7 ± 2.4 µg/mL. Previous studies have also indicated their bactericidal properties against various pathogenic microbial organisms [31,32,33]. Emerging studies have also indicated that chalcones and their derivatives prevent biofilm formation in bacterial colonies [34,35,36] and enhance antibiotic sensitivity [37,38]. The bacterial strains used are pathogenic to humans and animals as well as known to be lethal in many conditions [39,40]. The infection of *P. aeruginosa* has been widely associated with patients of cancers, organ transplantation and HIV [41]. Additionally, there have also been raising concerns about antibiotic resistance over the years. Likewise, *S. aureus* and *S. enteritidis* are also associated with infections of the digestive tract in foodborne diseases [42,43]. Hence, the inhibitory potential of bis-chalcones on various microbes may indicate their potential as antibiotic agents for future use.

### 2.4. Larvicidal Activity of Synthetic Bis-Chalcones

Apart from their antioxidant properties, it was also noted that the bis-chalcones exhibited larvicidal properties. The larvicidal property of the synthetic bis-chalcones revealed stronger activity in compound **1** (45.27 ± 2.34 µg/mL), compound **2** (59.81 ± 2.09 µg/mL), and compound **4** (56.46 ± 3.07 µg/mL) (Table 3). Limited studies are available on the potential of synthetic chalcones or their derivatives against mosquito larvae. Previous studies by Targanski et al. [44], Begum et al. [45] and Pasquale et al. [46] are among the few that indicated the potential of chalcones against *Aedes aegypti.* Mosquitoes are important vectors of various diseases including arboviral diseases [47], Chikungunya [48], and dengue [49]. Further, the recent literature has indicated that mosquitoes are also involved in the spreading of Zika viral infections [50]. Therefore, the beneficial larvicidal potential of synthetic bis-chalcones may be helpful in the management of infectious diseases.

The cytotoxicity evaluation was performed against two human breast cancer cells, MCF-7 and MDA-MB-231 (Table 4). The MCF-7 cell expresses receptors for estrogen, epidermal growth factor, and progesterone [51,52,53]. On the contrary, MDA-MB-231 is a cell with basal expression for these three receptors and is often considered to be “triple negative” [54,55,56]. The chalcones were more toxic towards the MCF-7 cells compared to MDA-MB-231 in terms of the IC_50_ values. Previous studies have also reported the anticancer potentials of chalcones and their derivatives in various cell and animal models [57,58]. In addition, the fact that the synthetic chalcones are in a purified form means their applicability in medicine is higher than that of crude extracts or isolated plant products.

Further studies are also necessary to ascertain the safety aspects of these synthetic bis-chalcones against various non-target organisms, freshwater fishes and germinating seeds. This will ensure a bis-chalcone-based synthetic pesticide, which is an efficient alternative to the existing antioxidant supplement, antimicrobial compounds, and larvicidal systems. Additionally, further studies on the functional food aspect of the compounds will ensure the possible potential of these selected compounds as a nutraceutical against various diseases.

## 3. Materials and Methods

### 3.1. Chemicals and Reagents

The chemicals, reagents, and analytical-grade solvents were obtained from Sigma-Aldrich and local chemical companies. Bulk solvents used for purification or extraction and other general purposes were purified and dried before use by following standard procedures. Unless otherwise specified, chemicals or reagents were used as received from the suppliers without further purification. Chromatography was performed using Silica gel (60–120 and 100–200 mesh size). The progress and completion of the reaction were monitored by thin-layer chromatography (TLC). For this purpose, aluminum sheets pre-coated with silica gel (Merck, Kieselgel60, F254) were used.

### 3.2. Instruments Used for the Study

IR spectra were recorded on a Nicolet iS5 Thermo Fischer Scientific FT-IR spectrometer (Waltham, MA, USA). The spectral positions are given in the wavenumber (cm^−1^) unit. ^1^H and ^13^C NMR spectra of the compounds in CDCl_3_ were recorded using Bruker AMX-400 (400 MHz) spectrometer (Billerica, MA, USA). For ^1^H NMR spectra, the chemical shifts (δ) are reported in parts per million (ppm) relative to tetramethylsilane (TMS) as an internal standard. Coupling constants (*J*) are given in Hz. The spectrophotometric measurements were taken using UV 1280 Shimadzu UV/Visible spectrophotometer (Kyoto, Japan).

### 3.3. Synthesis and Characterization of Bis-Chalcones

**Figure 2:**
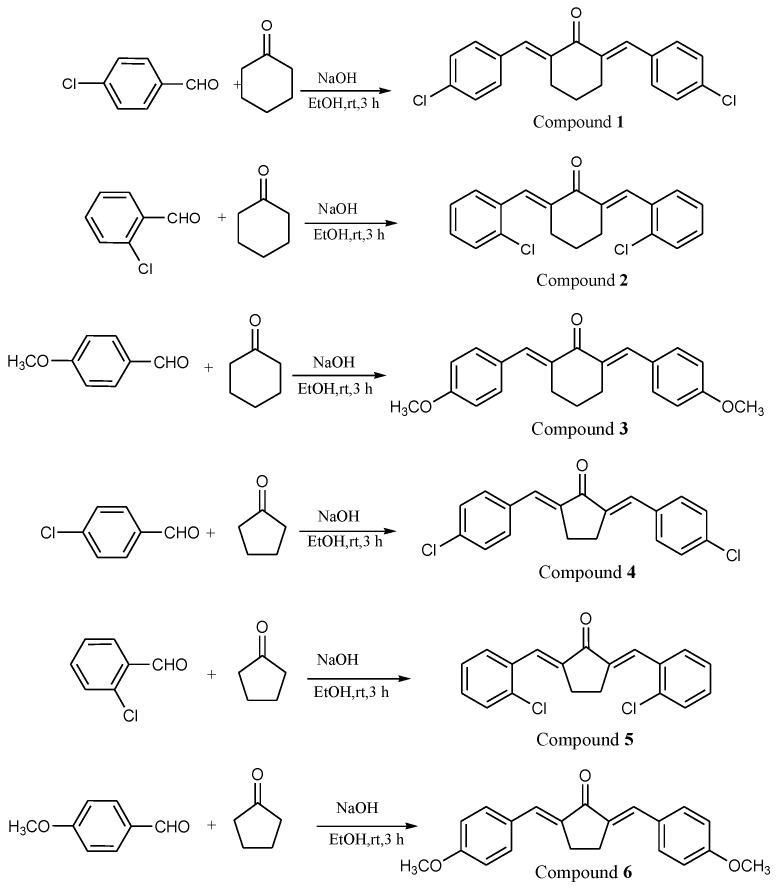
Synthetic route for the target compounds.

The target bis-chalcone compounds with cyclohexanone and cyclopentanone cores were synthesized as outlined in Figure 2. The required chemicals such as ortho-chloro benzaldehyde, para-chloro benzaldehyde, para-methoxy benzaldehyde, cyclohexanone and cyclopentanone were obtained from Aldrich and used without further purification. Solvents and other reagents were obtained from local sources. The base-catalyzed Claisen–Schmidt condensation reaction of substituted benzaldehydes with cyclohexanone and cyclopentanone yielded the target compounds. In a typical reaction, the double mixed-aldol condensation reaction between a ketone and substituted benzaldehyde compound was carried out [16]. The ketone has α-hydrogens (on both sides) and thus can be deprotonated to give a nucleophilic enolate anion. The alkoxide produced is protonated by the solvent, giving a β-hydroxy ketone, which undergoes base-catalyzed dehydration. The elimination process is particularly fast in this case because the alkene is stabilized by conjugation not only to the carbonyl but also to the benzene. In this synthesis, two equivalents of the substituted benzaldehyde compound were used such that the aldol condensation could occur on both sides of the ketone. The aldehyde carbonyl is more reactive than that of the ketone and therefore reacts rapidly with the anion of the ketone to give a β-hydroxy ketone, which easily undergoes base-catalyzed dehydration (Figure 2). The molecular structures of all the target compounds were confirmed by standard spectroscopic methods of analysis. Detailed synthetic procedures for the compounds along with their characterization data are given as supplementary information.

### 3.4. Radical Generation Inhibition and Reducing Potential of the Synthetic Bis-Chalcones

The antiradical activity of the synthetic bis-chalcones was estimated against various radical generators including DPPH, ABTS, and nitric oxide according to the standard protocols mentioned by Lalhminghlui and Jagetia [59]. The reducing potential was assessed as the ferric reduction ability of the compounds as described by Youn et al. [60].

### 3.5. Antibacterial Activity of the Synthetic Bis-Chalcones by Disc Diffusion Method

The antibacterial activity was estimated against *Escherichia coli, Pseudomonas aeruginosa, Staphylococcus aureus*, and *Salmonella enteritidis* strains. Briefly, the bacteria were grown in LB broth, and for the disc diffusion assay they were plated in an MHA agar plate (5 mm thickness). The plate was immersed with an 8 mm filter-paper disc corresponding to 20 μg/mL. The plates were incubated for 24 h in a bacteriological incubator and the inhibition zone was determined [61].

### 3.6. Analysis of the Larvicidal Activity of Synthetic Bis-Chalcones

The larvae of *Aedes albopictus* (third instar) were collected from the maintained culture; different concentrations of the synthetic bis-chalcones were added to glass jars (500 mL) and fifty larvae were put in each. The larvae were observed for 24 h for mortality. The percentage of death in each treatment was determined and the LC_50_ value was determined by probit analysis.

### 3.7. Cytotoxicity Analysis of Synthetic Bis-Chalcones

The MCF-7 and MDA-MB-231 cells were procured from the National Centre for Cell Science (Pune, Maharashtra). The cells were maintained according to the methods suggested by the supplier. The cytotoxicity was estimated using the MTT assay as described by the studies of Khanapure et al. [62]. The IC_50_ value was estimated using probit analysis using GraphPad Prism.

### 3.8. Statistical Analysis

The experimental results were initially sorted using spreadsheet software and statistical analysis was performed using GraphPad Prism version 7.0 (La Jolla, CA, USA). All the data were represented as mean ± SD for every experiment.

## 4. Conclusions

In this study, we synthesized six bis-chalcone compounds based on cyclohexanone and cyclopentanone cores. Their molecular structures were characterized by spectroscopic methods. The bis-chalones were different in their aromatic cores, functional group substitution, and position of substitutions. The compounds with a cyclohexanone core and –Cl substitution exhibited better antioxidant properties. In addition, compounds **1**, **2**, and **4** exhibited strong antibacterial activities against *Escherichia coli, Pseudomonas aeruginosa, Staphylococcus aureus*, and *Salmonella enteritidis*. Apart from their antioxidant and antibacterial properties, the compounds also exhibited larvicidal properties. The effective control of mosquito populations by killing the larvae of the *Aedes* mosquito indicates their potential application in preventing infectious diseases and their vectors. The cytotoxic effect of these compounds is also indicative of their antineoplastic potentials. Our preliminary studies highlight the potential of bis-chalcones as pharmacologically active compounds and therefore further research with structural modifications with polar and non-polar substitutions is currently underway.

## Figures and Tables

**Table 1:** Antiradical properties of different synthetic bis-chalcones.

	DPPH (IC_50_ µg/mL)	ABTS (IC_50_ µg/mL)	Nitric Oxide (IC_50_ µg/mL)	FRAP (EC_50_ µg/mL)
(2E,6E)-2,6-bis(4-methoxybenzylidene) cyclohexanone (compound **1**)	18.41 ± 1.45	18.63 ± 1.41	28.87 ± 1.49	1.35 ± 0.10
(2E,6E)-2,6-bis(4-chlorobenzylidene) cyclohexanone (compound **2**)	19.92 ± 1.52	21.57 ± 1.55	26.04 ± 1.61	5.24 ± 0.21
(2E,6E)-2,6-bis(2-chlorobenzylidene) cyclohexanone (compound **3**)	27.75 ± 2.50	26.47 ± 1.42	34.30 ± 2.55	12.40 ± 0.20
(2E,5E)-2,5-bis(4-(tetrahydro-2H-pyran-2-yloxy) benzylidene) cyclopentanone (compound **4**)	25.42 ± 1.39	22.18 ± 1.29	29.15 ± 1.72	4.34 ± 0.11
2,5-bis(4-hydroxybenzylidene) cyclopentanone (compound **5**)	36.49 ± 1.55	42.10 ± 2.27	45.67 ± 3.04	15.61 ± 0.30
4-(tetrahydro-2H-pyran-2-yloxy) benzaldehyde (compound **6**)	35.47 ± 1.64	46.17 ± 3.23	49.09 ± 3.11	16.20 ± 0.24

**Table 2:** Efficacy of synthetic bis-chalcones against Gram-positive and Gram-negative bacterial strains as indicated by the zone of inhibition by the disc diffusion method.

Bacteria	Compounds with Zone of Inhibition (mm)					
	1	2	3	4	5	6
*Escherichia coli*	18.4 ± 0.1	22.5 ± 0.2 *	16.7 ± 0.3	20.6 ± 0.3 *	17.7 ± 0.3	16.4 ± 0.1
*Pseudomonas aeruginosa*	19.8 ± 0.2 *	19.1 ± 0.2 *	14.7 ± 0.2	20.1 ± 0.3	16.9 ± 0.2	15.0 ± 0.3
*Staphylococcus aureus*	18.5 ± 0.1	20.1 ± 0.3 *	13.7 ± 0.1	19.5 ± 0.2 *	15.1 ± 0.2	14.6 ± 0.3
*Salmonella enteritidis*	18.2 ± 0.1	19.0 ± 0.3 *	15.4 ± 0.2	18.6 ± 0.1 *	16.0 ± 0.2	17.4 ± 0.1

**Table 3:** Larvicidal properties of different synthetic bis-chalcones against *Aedes albopictus*.

Compound	LC_50_ (µg/mL)
(2E,6E)-2,6-bis(4-methoxybenzylidene) cyclohexanone (compound **1**)	45.27 ± 2.34
(2E,6E)-2,6-bis(4-chlorobenzylidene) cyclohexanone (compound **2**)	59.81 ± 2.09
(2E,6E)-2,6-bis(2-chlorobenzylidene) cyclohexanone (compound **3**)	99.04 ± 2.18
(2E,5E)-2,5-bis(4-(tetrahydro-2H-pyran-2-yloxy) benzylidene) cyclopentanone (compound **4**)	56.46 ± 3.07
2,5-bis(4-hydroxybenzylidene) cyclopentanone (compound **5**)	89.22 ± 3.12
4-(tetrahydro-2H-pyran-2-yloxy) benzaldehyde (compound **6**)	79.18 ± 2.69

**Table 4:** Cytotoxicity evaluation of the synthetic bis-chalcones against human cancer cells and results are expressed as IC_50_ values.

Compound	LC_50_ (µg/mL)	
	MCF-7	MDA-MB-231
(2E,6E)-2,6-bis(4-methoxybenzylidene) cyclohexanone (compound **1**)	86.13 ± 3.45	128.66 ± 3.62
(2E,6E)-2,6-bis(4-chlorobenzylidene) cyclohexanone (compound **2**)	79.51 ± 2.85	97.64 ± 3.15
(2E,6E)-2,6-bis(2-chlorobenzylidene) cyclohexanone (compound **3**)	132.49 ± 3.71	160.54 ± 5.22
(2E,5E)-2,5-bis(4-(tetrahydro-2H-pyran-2-yloxy) benzylidene) cyclopentanone (compound **4**)	71.09 ± 2.34	89.62 ± 2.18
2,5-bis(4-hydroxybenzylidene) cyclopentanone (compound **5**)	103.56 ± 2.48	141.05 ± 4.84
4-(tetrahydro-2H-pyran-2-yloxy) benzaldehyde (compound **6**)	109.82 ± 4.10	155.32 ± 5.03

## Data Availability

The data may be shared upon valid request.

## Supplementary Materials

The following supporting information can be downloaded at: https://www.mdpi.com/article/10.3390/molecules27238209/s1, Supplementary Figure S1: FT-IR spectrum of compound **1**; Supplementary Figure S2: ^1^H NMR Spectrum of compound **1** in CDCl3; Supplementary Figure S3: FT-IR spectrum of compound **2**; Supplementary Figure S4: ^1^H NMR Spectrum of compound **2** in CDCl3; Supplementary Figure S5: FT-IR spectrum of compound **3**; Supplementary Figure S6: ^1^H NMR Spectrum of compound **3** in CDCl3; Supplementary Figure S7: FT-IR spectrum of compound **4**; Supplementary Figure S8: ^1^H NMR Spectrum of compound **4** in CDCl3; Supplementary Figure S9: FT-IR spectrum of compound **5**; Supplementary Figure S10: ^1^H NMR Spectrum of compound **5** in CDCl3; Supplementary Figure S11: 13C NMR Spectrum of compound **5** in CDCl3; Supplementary Figure S12: FT-IR spectrum of compound **6**; Supplementary Figure S13: ^1^H NMR Spectrum of compound **6** in CDCl3.

## Sample Availability

Samples of the compounds are available from the authors.
