# Supplementary material for: Antioxidant, Antimicrobial, Cytotoxicity, and Larvicidal Activities of Selected Synthetic Bis-Chalcones
Source: Molecules. 2022 Nov 25;27(23):8209. doi: 10.3390/molecules27238209 (PMC9740027; doi:10.3390/molecules27238209)
Supplement: Supplementary file 1 [file molecules-27-08209-s001.zip › molecules-2001710-supplementary.pdf]

Synthesis of Compound 1

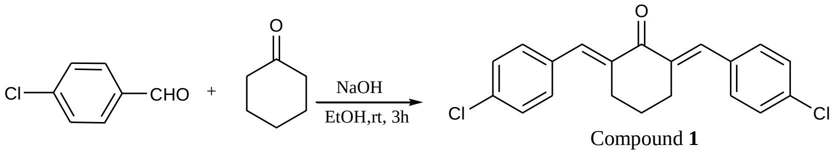

Compound **1**

To a stirring solution of cyclohexanone (1 equivalent) and *p*-chlorobenzaldehyde (2 equivalent) in ethanol was added a solution of 10% NaOH in ethanol. The resulting solution was allowed to stir at room temperature. The reaction mixture was cooled in an ice bath, and the product was collected by suction filtration on the Buchner funnel. Mixed solvent recrystallization from DCM-EtOH afforded compound 1 as yellow crystals in 66% yield. MP: 220-225 °C. R_f =0.66 in 20% EtOAc – Hexane, IR (ν cm^{-1}): 3058, 2963, 1670, 1605, 1575, 1488, 1401, 1319, 1264, 1091, 977, 929, 835, 821, 798, 524.

^{1}H NMR (500 MHz, $CDCl_3$): δ ppm 7.7(s, 2H, Olefinic-H),7.35 (t, 8H, Ar-H), 2.86 - 2.84 (m, 4H, 2xCH_2), 1.81-1.74 (m, 2H, 1xCH_2).

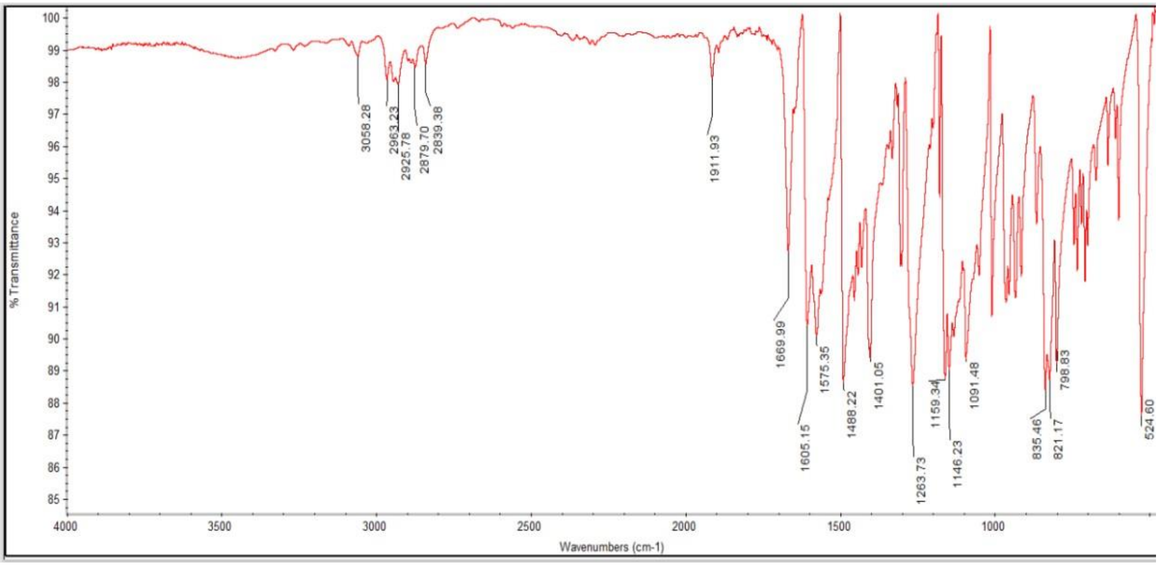

Supplementary Figure S1. FT-IR spectrum of compound **1**

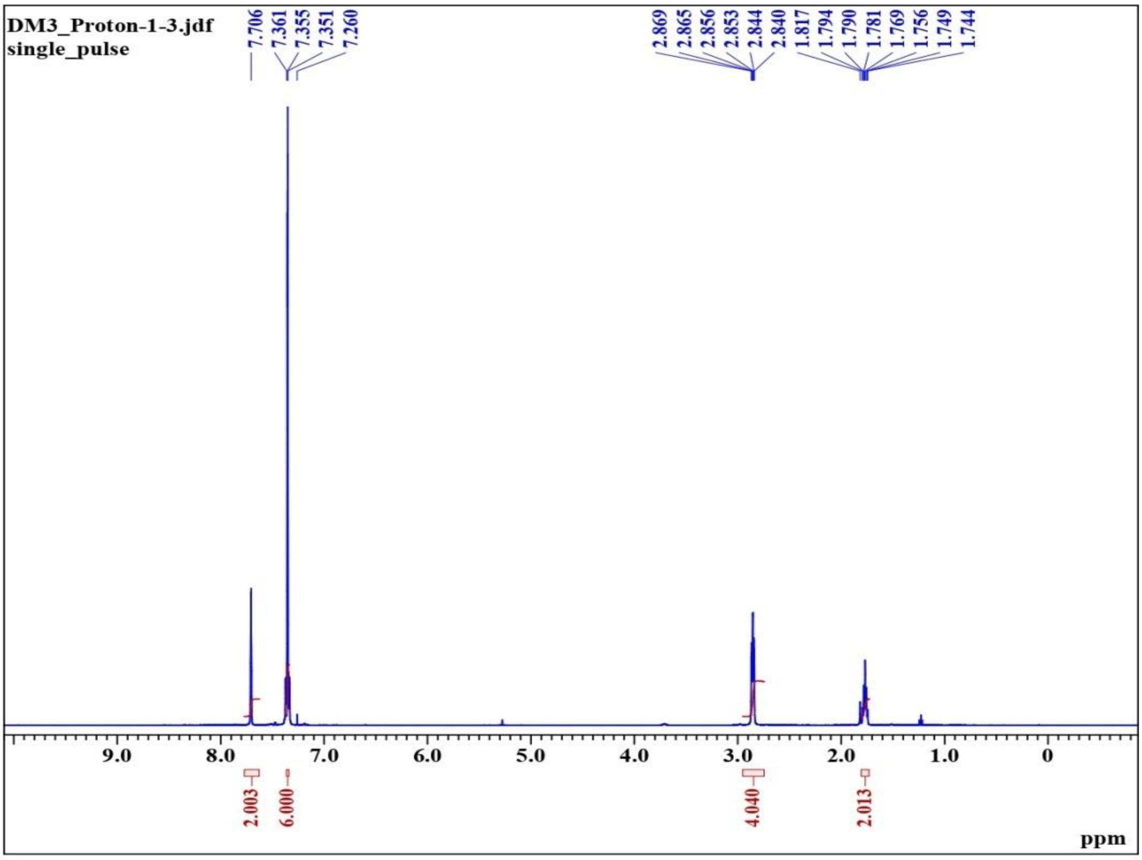

Supplementary Figure S2. ^{1}H NMR Spectrum of compound **1** in $CDCl_3$

Synthesis of Compound 2

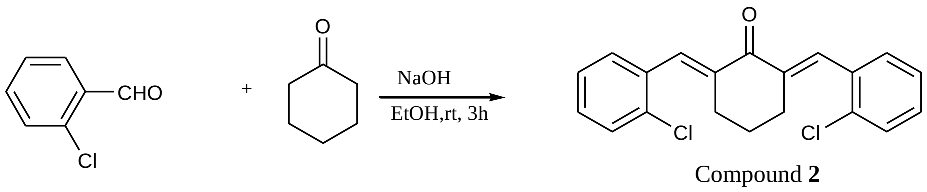

Compound **2**

To a stirring solution of cyclohexanone (1 equivalent) and *o*-chlorobenzaldehyde (2 equivalent) in ethanol was added a solution of 10% NaOH in ethanol. The resulting solution was allowed to stir at room temperature. The reaction mixture was cooled in an ice bath, and the product was collected by suction filtration on Buchner funnel. A mixed solvent recrystallisation from DCM−EtOH afforded compound **2** as yellow crystals in 90.6 % yield. MP: 220-225 ^{o}C. R_f =0.4883 in 20% EtOAc – Hexane.

IR (ν cm^{-1}): 3070, 2970, 1663,1601, 1575, 1467, 1432, 1260, 1169, 1145, 1119.5, 977, 929, 835, 821, 766, 735.

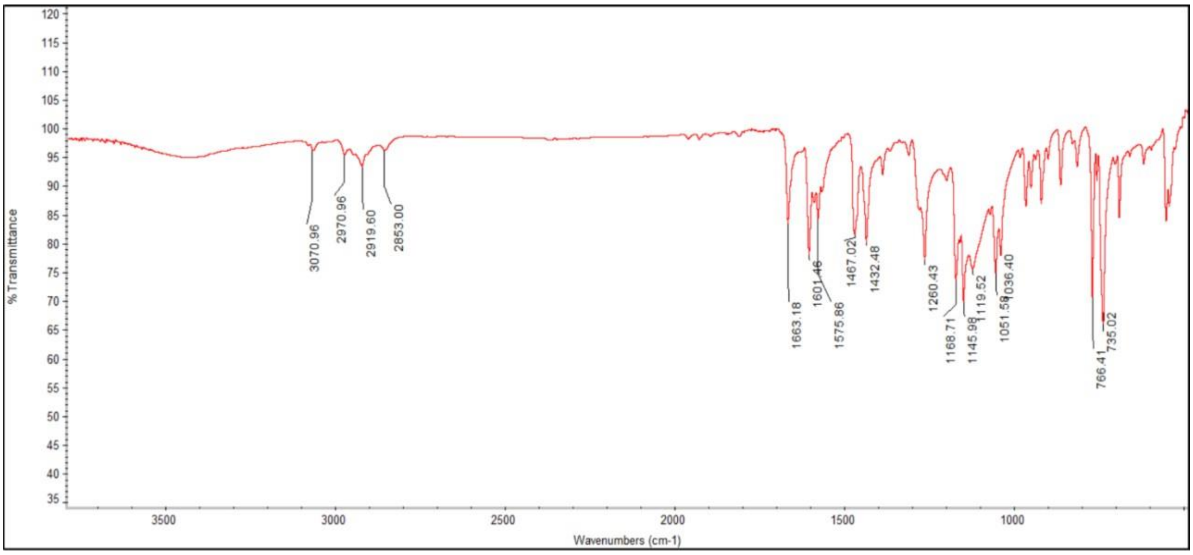

Supplementary Figure S3. FT-IR spectrum of compound **2**

^{1}H NMR (500 MHz, $CDCl_3$): δ ppm 7.90 (s, 2H, Olefinic-H), 7.43-7.41(m, 2H, Ar-H), 7.33-7.31(m, 2H, Ar-H), 7.28-7.25(m, 4H, Ar-H), 2.75(t, 4H, J=5Hz, 2xCH_2), 1.34 (m, 2H, 1xCH_2).

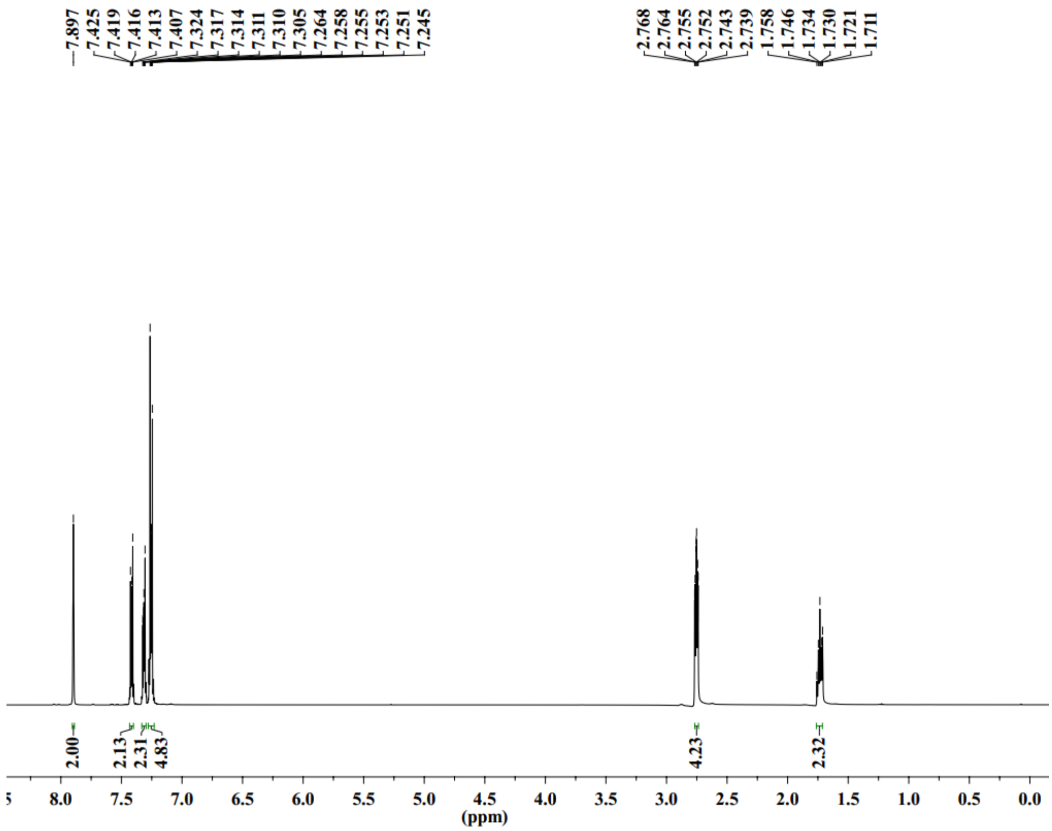

Supplementary Figure S4. ^{1}H NMR Spectrum of compound **2** in $CDCl_3$

Synthesis of Compound 3

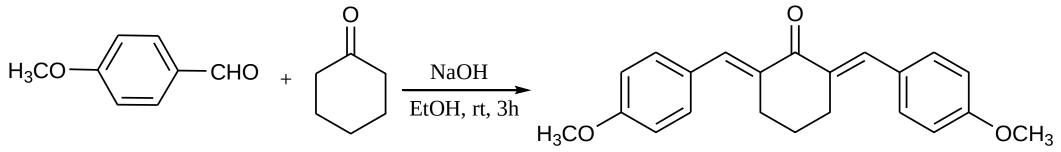

Compound **3**

To a stirring solution of cyclohexanone (1 equivalent) and *p*-methoxybenzaldehyde (2 equivalent) in ethanol was added a solution of 10% NaOH in ethanol. The resulting solution was allowed to stir at room temperature. The reaction mixture was cooled in an ice bath, and the product was collected by suction filtration on Buchner funnel. Mixed solvent recrystallization from DCM–EtOH afforded compound **3** as yellow crystals in 48.8% yield. R_f =0.73 in 20% EtOAc – Hexane.

IR (ν cm^{-1}): 2963, 2916, 2843, 2051, 1915, 1696, 1616, 1597, 1508, 1438, 1418, 1365, 1305, 1253, 1171, 1119, 1029, 987, 929, 835, 819, 689, 607, 536, 517. ^{1}H NMR (500 MHz, $CDCl_3$): δ ppm 7.76 (s, 2H,Olefinic-H), 7.46 – 7.44 (m,4H, Ar-H), 6.94 - 6.92 (m, 4H, Ar-H), 3.83 (s, 6H, 2 x $-OCH_3$), 1.8 - 1.6 (m, 2H, 1xCH_2), 2.92 -2.89 (m, 4H, 2xCH_2).

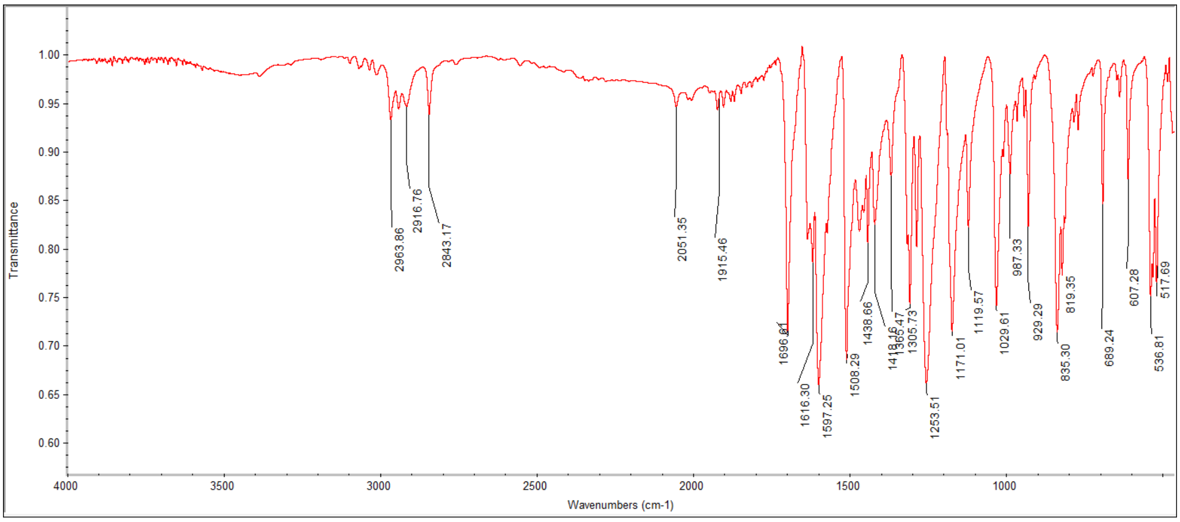

Supplementary Figure S5. FT-IR spectrum of compound **3**

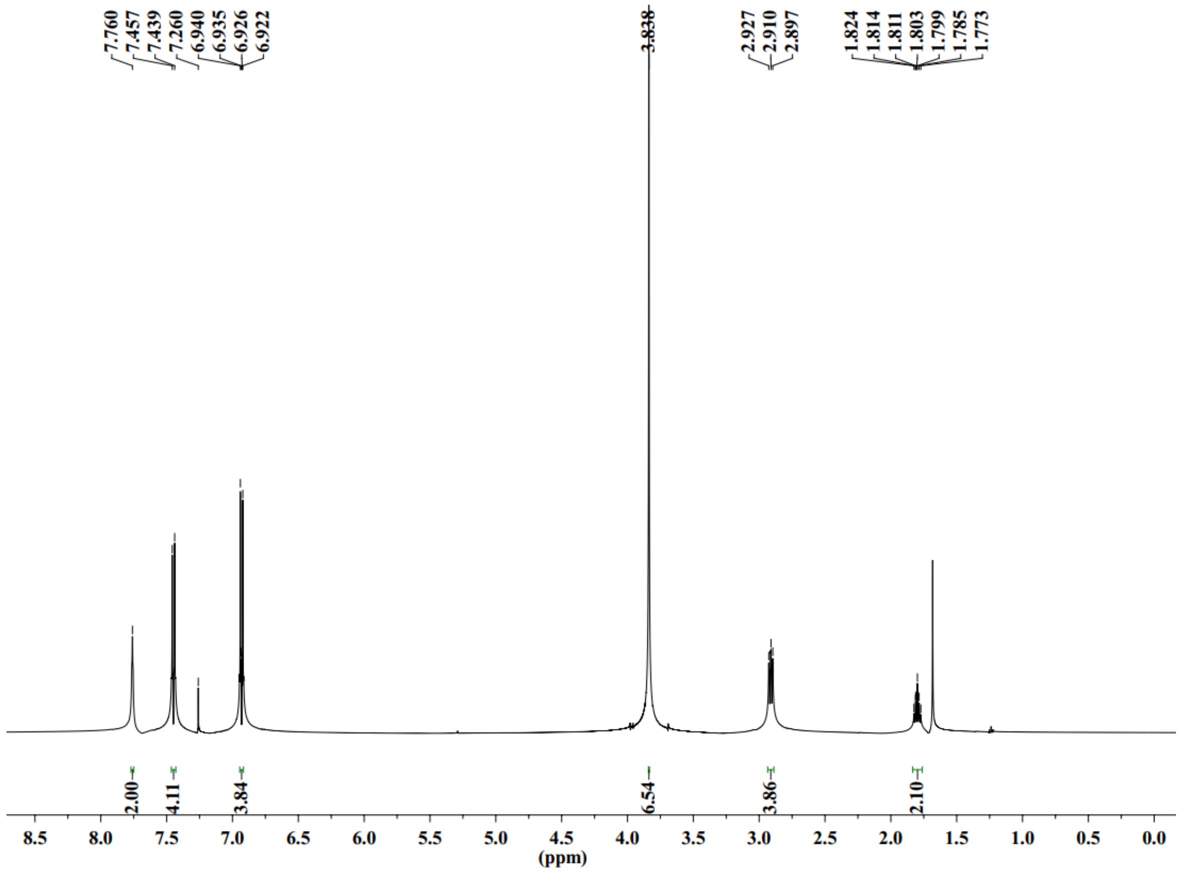

Supplementary Figure S6. ^{1}H NMR Spectrum of compound **3** in $CDCl_3$

Synthesis of Compound 4

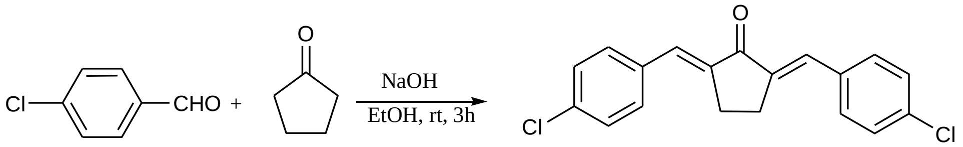

Compound **4**

To a stirring solution of cyclopentanone (1 equivalent) and *p*-chlorobenzaldehyde (2 equivalent) in ethanol was added a solution of 10% NaOH in ethanol. The resulting solution was allowed to stir at room temperature. The reaction mixture was cooled in an ice bath, and the product was collected by suction filtration on the Buchner funnel. Mixed solvent recrystallization from DCM−EtOH afforded compound 4 as yellow crystals in 49.4% yield. R_f =0.58 in 20%. EtOAc – Hexane, IR(ν cm^{-}1) 3378, 2919, 2366, 2342, 1913, 1694, 1621, 1607, 1584, 1556, 1489, 1404, 1306, 1278, 1253, 1178, 1106, 1092, 1009, 985, 929, 833, 820, 729, 685, 611, 520. ^{1}H NMR (500 MHz, $CDCl_3$): δ ppm 7.53-7.40 (m, 6 H, J = 8.5Hz, 2H (Olefinic-H), 4H (Ar-H), 7.34 (d ,4H, J = 8.5 Hz, Ar-H), 3.01 (s, 4H, 2xCH_2).

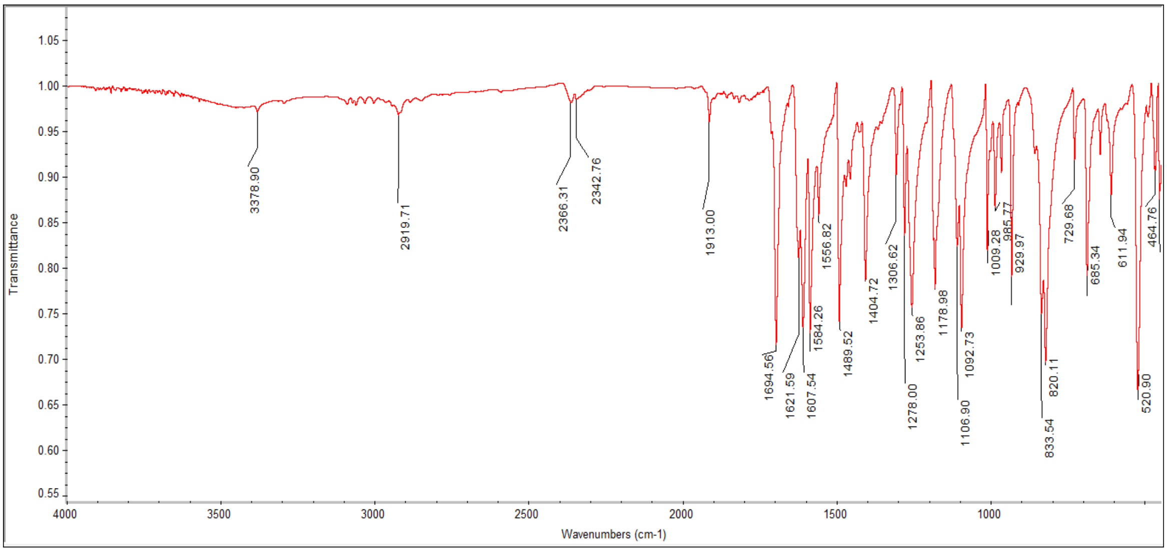

Supplementary Figure S7. FT-IR spectrum of compound **4**

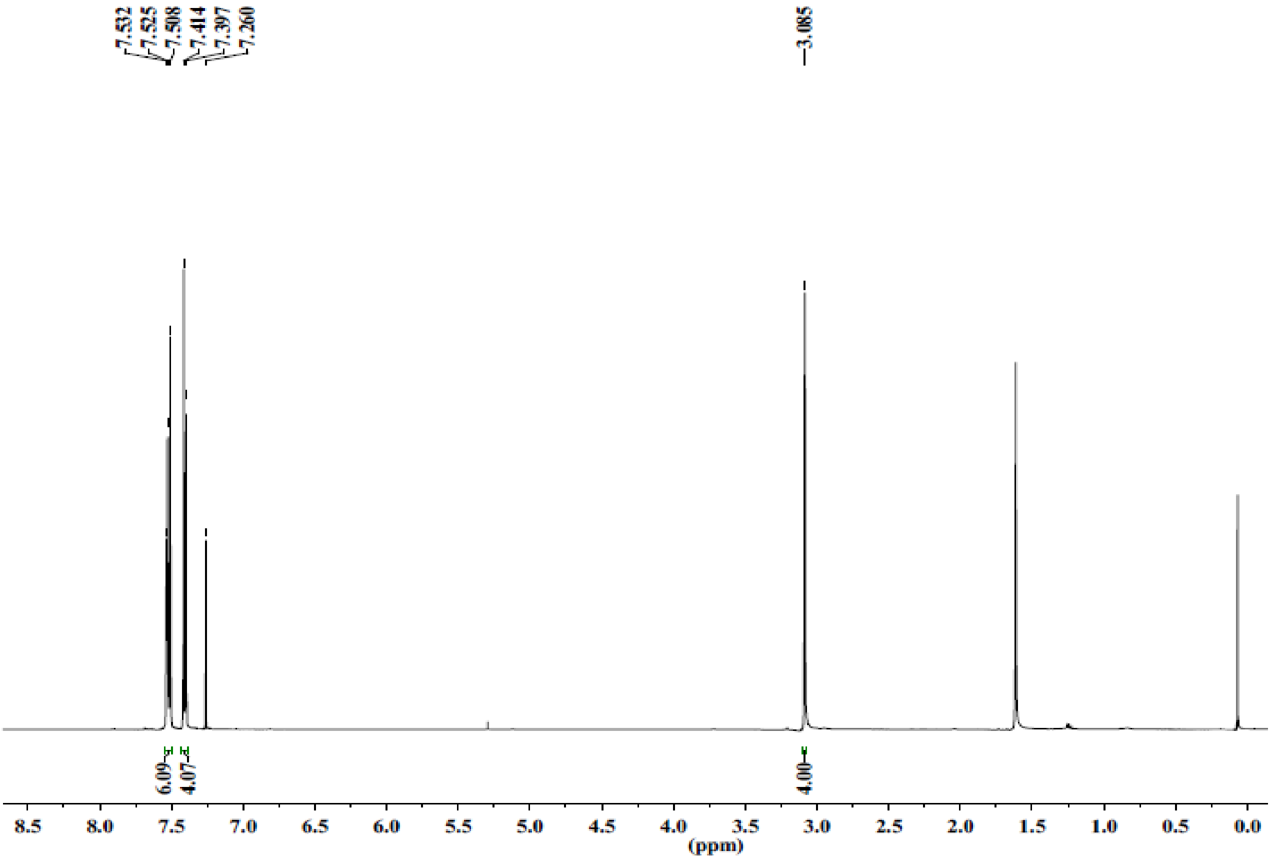

Supplementary Figure S8. ^1H NMR Spectrum of compound **4** in $CDCl_3$

Synthesis of compound 5

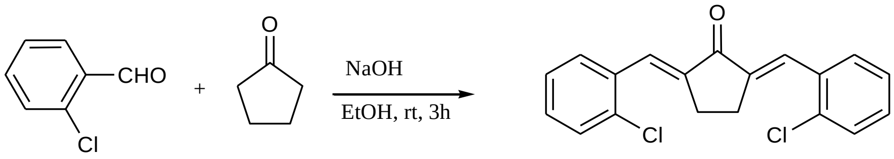

Compound **5**

To a stirring solution of cyclopentanone (1 equivalent) and *o*-chlorobenzaldehyde (2 equivalent) in ethanol was added a solution of 10% NaOH in ethanol. The resulting solution was allowed to stir at room temperature. The reaction mixture was cooled in an ice bath, and the product was collected by suction filtration on Buchner funnel. A mixed solvent recrystallisation from DCM−EtOH afforded compound **5** as yellow crystals in 69.5% yield.

R_f =0.51 in 20% EtOAc – Hexane, IR (ν cm^{-1}): 3069, 2912, 2361, 1687, 1620, 1599, 1587, 1560, 1465, 1449, 1431, 1277, 1239, 1182, 1155, 1124, 1038, 987, 941, 759, 751, 726, 688, 622, 544, 532. ^1H NMR (500 MHz, $CDCl_3$): δ ppm 7.90 (s, 2H, Olefinic-H), 7.53 – 7.42 (m, 4H, Ar-H), 7.30 -7.27 (d, 4H, J = 1.5 Hz, Ar-H), 2.97 (s, 4H, 2 x CH_2).

^{13}C NMR (500 MHz, $CDCl_3$): δ ppm 195.57, 139. 36, 136.13, 133.91, 130.30, 130. 19, 130. 13, 126.71, 26.61.

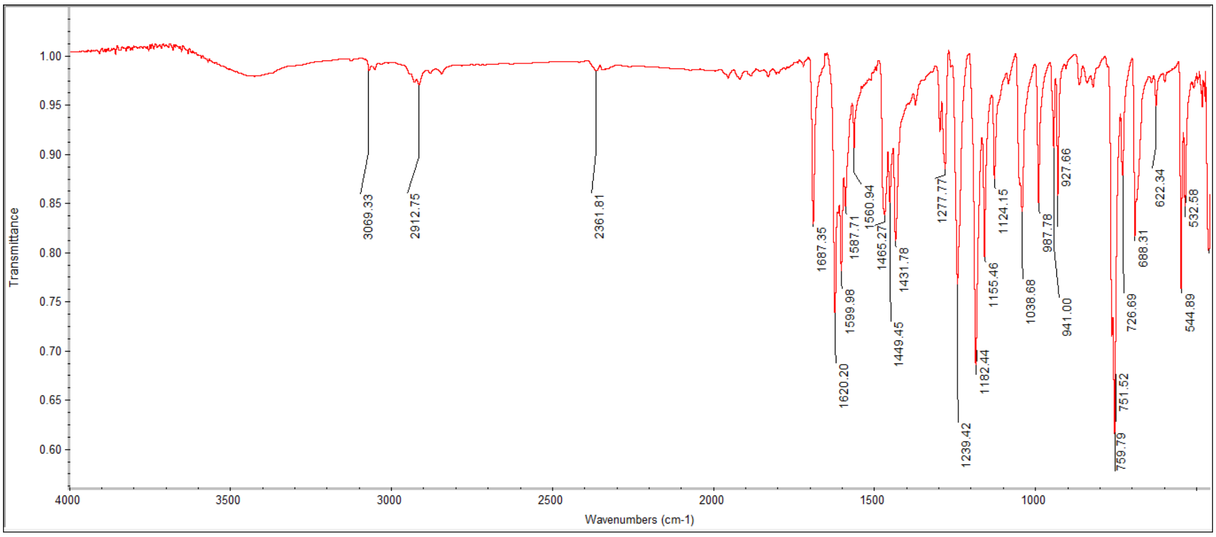

Supplementary Figure S9. FT-IR spectrum of compound **5**

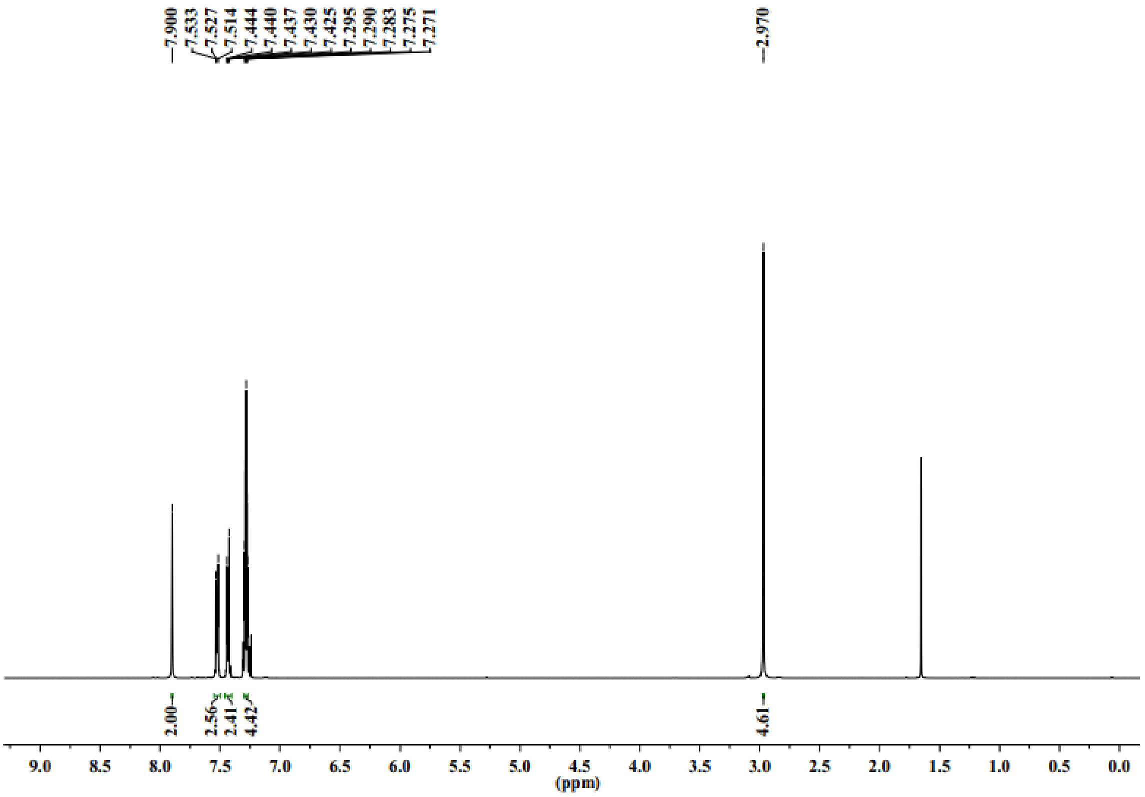

Supplementary Figure S10. ^{1}H NMR Spectrum of compound **5** in $CDCl_3$

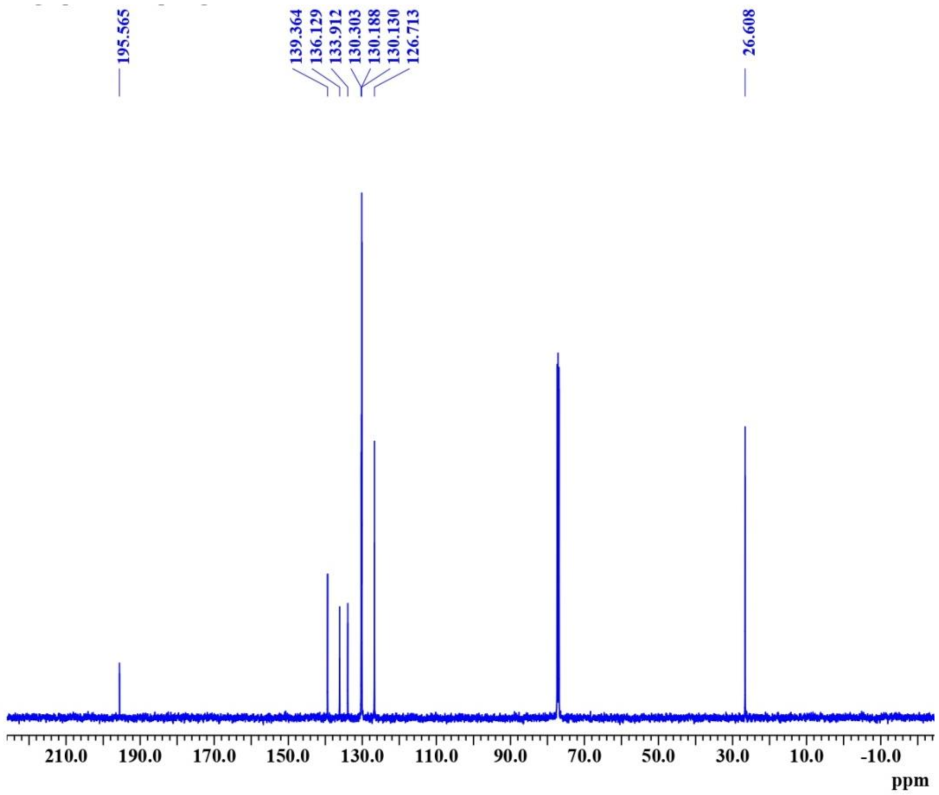

Supplementary Figure S11. ^{13}C NMR Spectrum of compound **5** in $CDCl_3$

Synthesis of compound 6

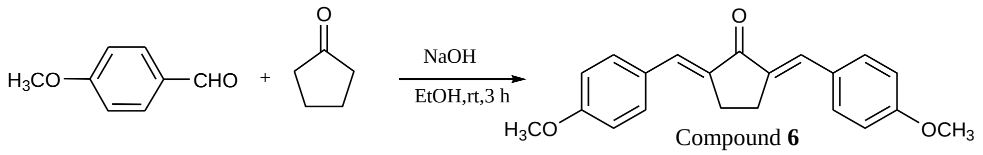

To a stirring solution of cyclopentanone (1 equivalent) and *p*-methoxybenzaldehyde (2 equivalent) in ethanol was added a solution of 10% NaOH in ethanol. The resulting solution was allowed to stir at room temperature. The reaction mixture was cooled in an ice bath, and the product was collected by suction filtration on the Buchner funnel. Mixed solvent recrystallization from DCM−EtOH afforded compound **6** as yellow crystals in a 49.7 % yield. R_f =0.64 in 20% EtOAc – Hexane. IR (ν cm^{-1}): 3069, 2963, 2937, 2913, 2846, 2057, 2007, 1696, 1616, 1597, 1508, 1439, 1365, 1306, 1252, 1171, 1029, 929, 835, 819, 694, 608, 536, 517. ^{1}H NMR (500 MHz, $CDCl_3$): δ ppm 7.57 – 7.55 (m, 6H, Ar-H), 6.96−6.95 (m, 4H, Ar-H), 3.85 (s, 6H, 2 x OCH_3), 3.06 (s, 4H, 2xCH_2).

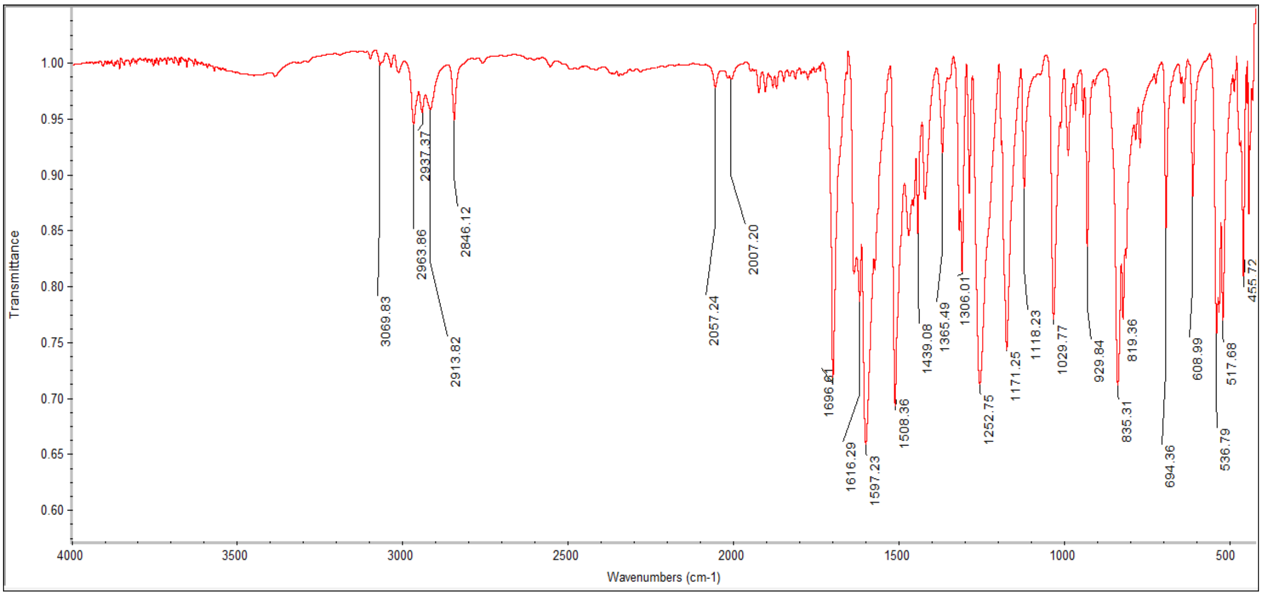

Supplementary Figure S12. FT-IR spectrum of compound **6**

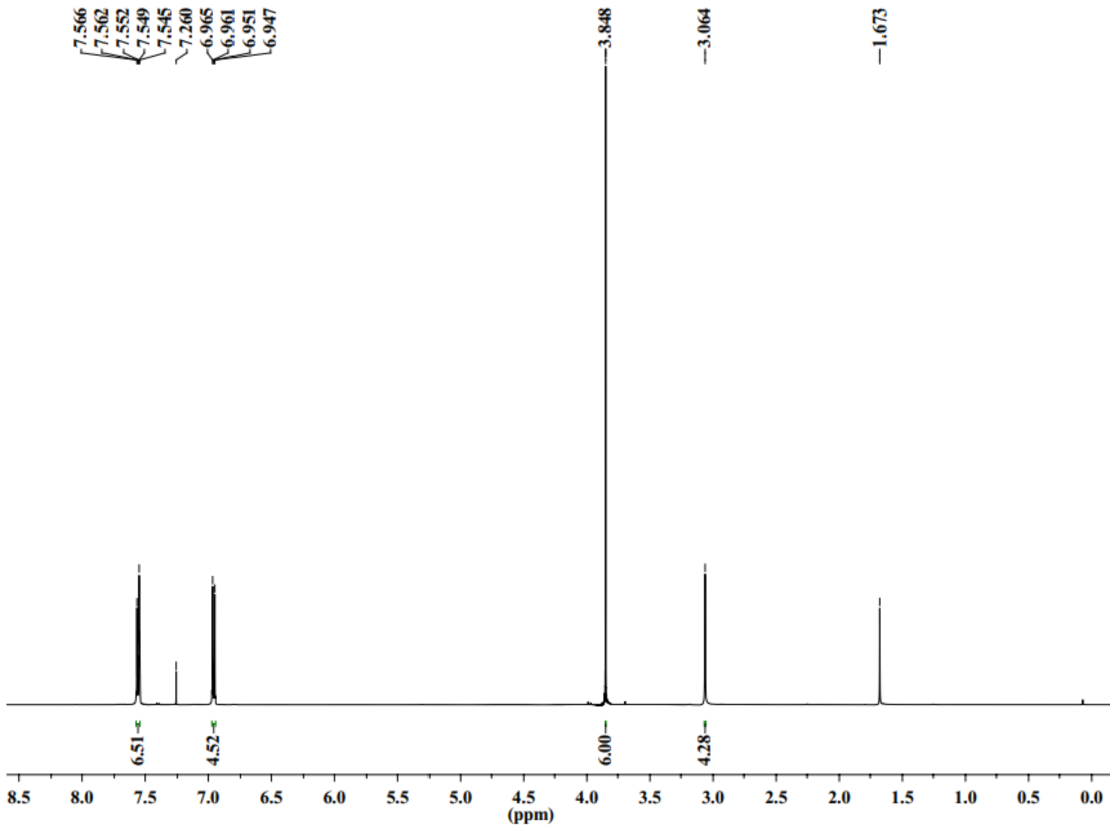

Supplementary Figure S13. ^{1}H NMR Spectrum of compound **6** in $CDCl_3$
